# Effect of subtherapeutic vs. therapeutic administration of macrolides on antimicrobial resistance in *Mannheimia haemolytica* and enterococci isolated from beef cattle

**DOI:** 10.3389/fmicb.2013.00133

**Published:** 2013-05-27

**Authors:** Rahat Zaheer, Shaun R. Cook, Cassidy L. Klima, Kim Stanford, Trevor Alexander, Edward Topp, Ron R. Read, Tim A. McAllister

**Affiliations:** ^1^Lethbridge Research Centre, Agriculture and Agri-Food CanadaLethbridge, AB, Canada; ^2^Alberta Agriculture and Rural DevelopmentLethbridge, AB, Canada; ^3^Southern Crop Protection and Food Research Centre, Agriculture and Agri-Food CanadaLondon, ON, Canada; ^4^Faculty of Medicine, University of CalgaryCalgary, AB, Canada

**Keywords:** macrolides, antimicrobial resistance, *Mannheimia haemolytica*, enterococci, beef cattle

## Abstract

Macrolides are the first-line treatment against bovine respiratory disease (BRD), and are also used to treat infections in humans. The macrolide, tylosin phosphate, is often included in the diet of cattle as a preventative for liver abscesses in many regions of the world outside of Europe. This study investigated the effects of administering macrolides to beef cattle either systemically through a single subcutaneous injection (therapeutic) or continuously in-feed (subtherapeutic), on the prevalence and antimicrobial resistance of *Mannheimia haemolytica* and *Enterococcus* spp. isolated from the nasopharynx and faeces, respectively. Nasopharyngeal and faecal samples were collected weekly over 28 days from untreated beef steers and from steers injected once with tilmicosin or tulathromycin or continuously fed tylosin phosphate at dosages recommended by manufacturers. Tilmicosin and tulathromycin were effective in lowering (*P* < 0.05) the prevalence of *M. haemolytica*, whereas subtherapeutic tylosin had no effect. *M. haemolytica* isolated from control- and macrolide-treated animals were susceptible to macrolides as well as to other antibiotics. Major bacteria co-isolated with *M. haemolytica* from the nasopharynx included *Pasteurella multocida*, *Staphylococcus* spp., *Acinetobacter* spp., *Escherichia coli* and *Bacillus* spp. With the exception of *M. haemolytica* and *P. multocida*, erythromycin resistance was frequently found in other isolated species. Both methods of macrolide administration increased (*P* < 0.05) the proportion of erythromycin resistant enterococci within the population, which was comprised almost exclusively of *Enterococcus hirae*. Injectable macrolides impacted both respiratory and enteric microbes, whereas orally administered macrolides only influenced enteric bacteria.

## Introduction

Bovine respiratory disease (BRD), commonly known as shipping fever continues to be one of the most economically significant health issues in feedlot cattle. The pathogenesis of BRD is multifactorial, being influenced by stress, immune status as well as viral/bacterial interactions within the respiratory tract. Regardless of what initiates the disease, *Mannheimia haemolytica* is considered to be the predominant bacterial pathogen associated with BRD (Confer, 2009).

To reduce or treat BRD, antibiotics are commonly administered to cattle upon arrival in North American feedlots. The use of antimicrobial therapy to control BRD increases in high-density feedlots where conditions are favorable for the introduction and transmission of infectious microbes. The macrolides, tilmicosin and tulathromycin are frequently administered subcutaneously to high-risk cattle, either prophylactically, metaphylactically, or therapeutically to cattle suffering from the disease. In North America, the macrolide tylosin phosphate is also included in beef cattle diets as a growth promoter and to prevent liver abscesses, a practice banned in Europe. After ionophores and tetracycline, macrolides are the most frequently used antimicrobials in cattle production in Canada (CIPARS, 2013). In the United States, a survey of 84% of the US feedlots revealed that about 42% of cattle received tylosin in feed for 138–145 days whereas over two-thirds of the cattle received injectable macrolides (USDA, 1999).

Macrolides belonging to the antimicrobial drug superfamily MLS_B_ (macrolide–lincosamide–streptogramin B) are classified as category II antimicrobials by the WHO and Health Canada (http://hc-sc.gc.ca/dhp-mps/vet/antimicrob/amr_ram_hum-med-rev-eng.php) emphasizing their importance in treating infections in humans. As reviewed by Gow (2005), as a proportion of total DDDs (Defined Daily Dose) for humans, after penicillins (27%), macrolides (20%) constitute the second most common systemic antibacterial class dispensed by retail pharmacies in Canada, followed by tetracyclines (14%), fluoroquinolones (12%), first- and second-generation cephalosporins (10%).

Although tilmicosin, tulathromycin and tylosin are exclusively used in food animals, they belong to the same category II MLS_B_ superfamily as erythromycin, which is used in both humans, food and companion animals. Despite having slight structural differences these drugs cross-select for resistance to all drugs of this superfamily, including several drugs used to treat infections in humans such as erythromycin and its derivatives azithromycin and clarithromycin (Roberts, 2008; Desmolaize et al., 2011). Consequently, use of macrolides in livestock could affect the efficacy of these antibiotics in controlling infections in humans through selection for resistance. Macrolide resistance can be conferred by discrete point mutations at nucleotide A2058 and its neighbours in the 23S rRNA, altering the main anchoring point for these antibiotics (Schlünzen et al., 2001) or by methylation of the A2058 at the N6 position as catalyzed by the Erm family of methyltransferases (Skinner et al., 1983). Drug efflux systems have also been shown to result in macrolide resistance (Roberts et al., 1999).

Enterococci are common members of the normal gut flora of both livestock and humans (Yost et al., 2011), but they can also be important human pathogens as *Enterococcus faecalis* and *Enterococcus faecium* are often implicated in nosocomial infections. Macrolide resistant enterococci have been isolated from cattle and depending on the species, could potentially colonize the intestinal tract of humans if they enter the food chain (Giraffa, 2002; Jensen et al., 2002).

The present study investigated and compared the response of respiratory and digestive tract bacteria in feedlot cattle to no antibiotic treatment or treatment with macrolide antibiotics at subtherapeutic (in-feed) or therapeutic (via injection) levels. Our specific objective was to evaluate the effects of administering macrolides to beef cattle either systemically through a single subcutaneous injection (tilmicosin and tulathromycin) or continuously in-feed (tylosin phosphate), on the prevalence and the antimicrobial resistance profiles of faecal *Enterococcus* spp., and *M. haemolytica* from the nasopharynx.

## Materials and methods

### Experimental design

**Figure 1:**
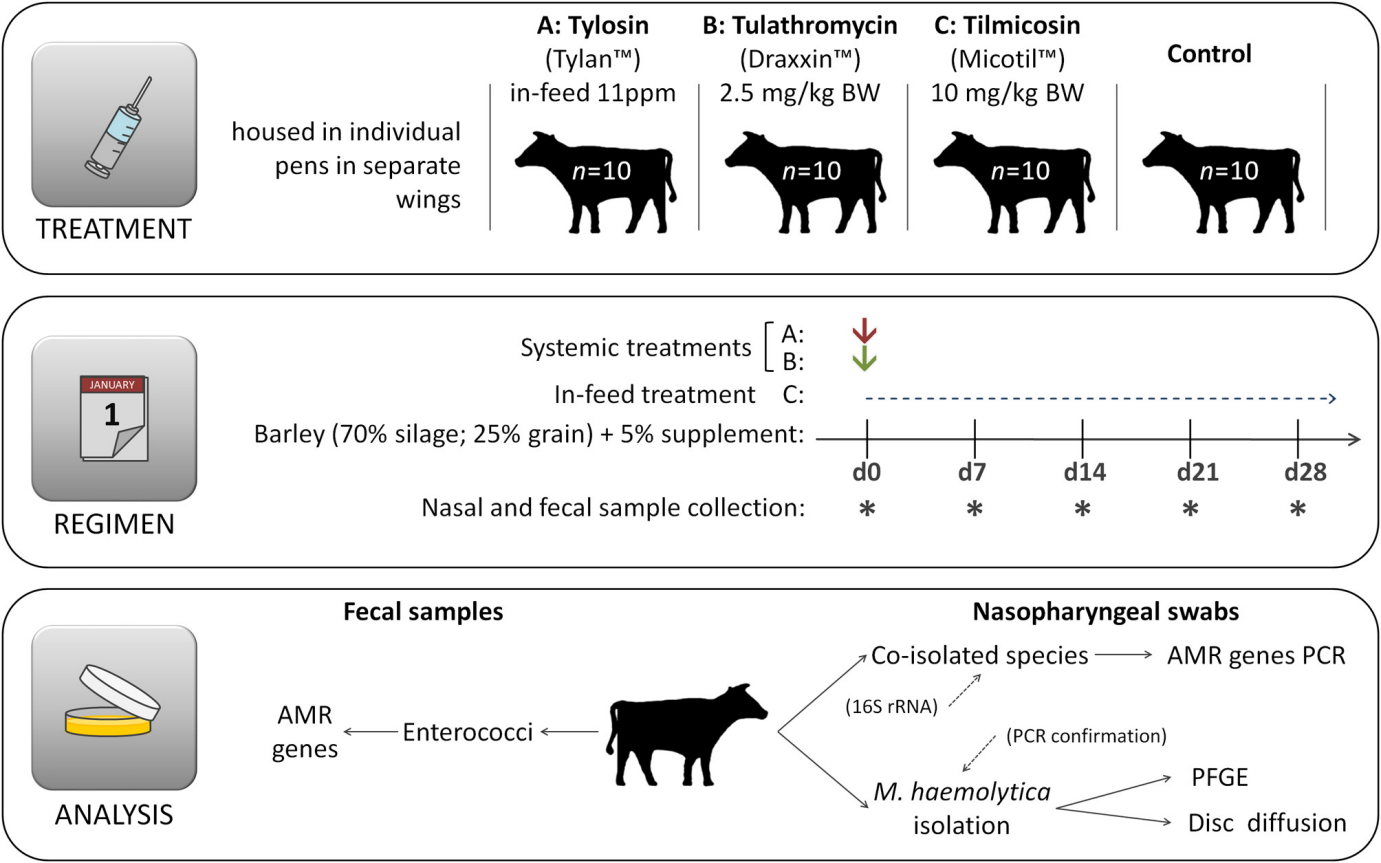
**Experimental design.** Immediately after first sample collection on day 0 (d0) animals were administered appropriate treatments and hence the same day is also referred as day 1, denoting the beginning of the experimental period. Cattle had no prior direct exposure to antibiotics prior to initiation of the experiment.

The study was conducted at the individual feeding barn facility at the Lethbridge Research Centre (Lethbridge, Alberta, Canada) using 40–eleven month old beef steers (394 ± 37 kg). All steers originated from the same ranch and had not received antibiotics during their lifetime prior to their arrival at the Lethbridge Research Centre. Steers were housed in individual pens with 10 replicate animals for each of the four treatments (1) control, no antibiotics; (2) tilmicosin (Micotil® Elanco Animal Health) single subcutaneous injection at 10 mg/kg bodyweight (BW) on day 1; (3) tulathromycin (Draxxin® Pfizer Animal Health, www.pfizer.ca) single subcutaneous injection at 2.5 mg/kg BW on day 1; (4) tylosin phosphate (Tylan®, Elanco Animal Health, www.elanco.ca) at 11 ppm in feed for the entire 28 day experimental period (Figure 1). Adjacent pens within the same treatment group shared a common water trough and cattle within each treatment were housed in separate, but otherwise identical wings of the barn. Throughout the study, care of the steers was in accordance with the guidelines set by the Canadian Council on Animal Care (http://www.ccac.ca/).

### Diet and feeding

Steers were housed in individual pens and fed a typical forage-based growing diet consisting of 70% barley silage, 25% barley grain, and 5% supplement (Addah et al., 2011) on a dry matter (DM) basis, for the entire experimental period (Figure 1). Steers were fed once daily in a manner that ensured that all feed that was allotted was consumed. To avoid cross contamination of feed, tylosin was mixed with 5 kg of supplement and manually spread over the surface of feed within each of the appropriate pens during the morning feeding. All cattle were provided feed at the same time each day with no feed remaining in the bunk prior to the next feeding. Cattle assigned to the control treatment had no access to medicated feed throughout the entire experiment and were not injected with any antibiotics.

### Sampling and sample processing

Rectal faecal and nasopharyngeal swab samples were taken from all 40 animals on arrival (day 0) at the facility, and then weekly thereafter for 4 weeks and processed as follows:

#### Faecal samples

Rectal grab faecal samples were taken and used for subsequent determination of antimicrobial resistant and total numbers of enterococci bacteria. For the isolation of *Enterococcus* species faecal samples (1 g) were diluted 1:5 in phosphate buffered saline, from which serial dilutions were made up to 10^−5^ and 100 μL of each dilution was spread-plated, in duplicate, onto Bile-Esculin-Azide (BEA) agar plates. Dilutions 10^−1^ and 10^−2^ were also spread-plated onto BEA agar containing erythromycin at a concentration of 8 μg/mL of media (BEA+Ery) to isolate macrolide-resistant enterococci. Plates were incubated at 37°C for 24 h and colonies from both BEA and BEA+Ery plates were enumerated. Three to five presumptive *Enterococcus* colonies per sample per media type were isolated and streak-purified onto BEA or BEA+Ery plates accordingly. Purified isolates were stored in glycerol stocks at −80°C until further characterized.

#### Nasopharyngeal swab samples

Nasopharyngeal swab samples were collected using a commercially available deep, double guarded culture swab (Jorgensen Laboratories, Inc., Loveland, CO, USA) from all 40 steers on arrival at the beef-barn facility prior to administration of antibiotics, and then weekly thereafter for 4 weeks following antibiotic treatment. Swab samples were transported to the lab on ice and immediately suspended in 0.7 mL of Brain Heart Infusion (BHI) broth. Aliquots (100 μL) were cultured at 37°C for 16 h onto BAC-agar plates (tryptic soy agar plates containing 5% sheep blood and 15 μg/mL of bacitracin; Dalynn Biologicals, Inc., Calgary, AB, Canada) with and without the addition of erythromycin (Ery) 8 μg/mL at 37°C for 16 h. Colonies (1–5) indicative of *Mannheimia*, were selected and tested for catalase and oxidase activity as described previously (Klima et al., 2011). Isolates that exhibited typical *M. haemolytica* colony morphology and were both catalase and oxidase positive were subsequently confirmed using a multiplex PCR assay (Alexander et al., 2008). Confirmed *M. haemolytica* isolates were stored at −80°C in BHI broth containing 20% glycerol for further characterization. Colonies that did not exhibit morphology indicative of *Mannheimia* on BAC or BAC+Ery agar plates were identified and 1–2 colonies representing each morphotype were selected and stored at −80°C in BHI broth containing 20% glycerol until further characterized. Bacitracin in BAC media inhibited the growth of the majority of gram positive bacteria, thereby improving the likelihood of isolating *M. haemolytica.* Although bacitracin resistant gram positive bacteria were co-isolated with *Mannheimia*, these isolates were subsequently identified using 16S rRNA profiling as described below.

### Characterization of *Mannheimia haemolytica* and co-isolated nasopharyngeal bacteria

Confirmed *M. haemolytica* isolates were serotyped as previously described (Klima et al., 2011) with antisera prepared in rabbits against formalin-killed whole cells of *M. haemolytica* reference strains UGCC G1 (serotype 1), UGCC G2 (serotype 2), ATCC 29697 (serotype 6), ATCC 29698 (serotype 7), and ATCC 29700 (serotype 9). Isolates were subject to pulsed field gel electrophoresis (PFGE) profiling using *Sal*I restriction enzyme as previously described (Klima et al., 2011). The 16S rRNA genes were PCR amplified from bacteria co-isolated with *M. haemolytica* using universal bacterial 16S rRNA gene primers 27F (5′-AGAGTTTGATCCTGGCTCAG-3′) and 1492R (5′-GGTTACCTTGTTACGACTT-3′) and subject to DNA sequencing (Eurofins MWG Operon, Huntsville, Alabama, USA) using one (27F) or both (27F and 1492R) primers.

### Antibiograms

Disk susceptibility tests were conducted for *Mannheimia* in accordance with the Clinical and Laboratory Standards Institute documents M31-A3 and M45-A (CLSI, 2008a,b). The antimicrobials tested, suppliers and resistance breakpoints applied are listed in Table 1. Reference strains *Escherichia coli* ATCC 35218, *Staphylococcus aureus* ATCC 25923, *Streptococcus pneumoniae* ATCC 49619, and *M. haemolytica* ATCC 33396 were used as quality controls. Briefly, cultures grown on Muller–Hinton agar supplemented with 5% defibrinated sheep blood (MHB; 16–18 h at 37°C) were suspended into Muller–Hinton broth to an absorbance reading between 0.125 and 0.145 at 625 nm. Using sterile swabs, the prepared inocula were swabbed onto MHB followed by the dispensation of the antibiotic containing disks onto the plate surface. The plates were incubated at 37°C in ambient air, with the exception of *S. pneumoniae* ATCC 49619 which required cultivation in a 5% CO_2_ atmosphere for 24 h. The resulting zones of inhibition were read using the BioMic V3 imaging system (Giles Scientific, Inc., Santa Barbara, CA, USA).

**Table 1:** **Antimicrobial agents, suppliers, disk contents, and interpretative criteria used for disk susceptibility testing**.

**Antimicrobial**	**Supplier**	**Supplier code**	**Disk content (μg)**	**Zone diameter (mm) breakpoints^c^**		
				**S**	**I**	**R**
Amoxicillin/clavulanic Acid^a^	BD	AMC-30	20/10	≥27	n/a	≤26
Ampicillin^a^	BD	AM-10	10	≥27	n/a	n/a
Ceftiofur^b^	BD	XNL-30	30	≥21	18–20	≤17
Danofloxacin^b^	Pfizer	DNO	5	≥22	n/a	n/a
Erythromycin^a^	BD	E-15	15	≥27	25–26	≤24
Florfenicol^b^	BD	FF-30	30	≥19	15–18	≤14
Gentamicin^b^	BD	GM-10	10	≥15	13–14	≤12
Oxytetracycline^a^	BD	T-30	30	≥23	n/a	n/a
Spectinomycin^b^	BD	SPT-100	100	≥14	11–13	≤10
Sulfamethoxazole^a^/trimethoprim	BD	SXT	23.75/1.25	≤24	n/a	n/a
Tilmicosin^b^	BD	TIL-15	15	≥14	11–13	≤10
Tulathromycin^b^	Pfizer	TUL	30	≥18	15–17	≤14

a M45-A: Methods for antimicrobial dilution and disk susceptibility testing of infrequently isolated or fastidious bacteria; approved guideline (CLSI, 2008b). Due to the unavailability MIC breakpoints for Mannheimia spp., guidelines for Pasteurella spp. were followed.

b M31-A3: Performance standard for antimicrobial disk and dilution susceptibility tests for bacteria isolated from animals; approved standard—third edition (CLSI, 2008a)

c Zone diameter value used to indicate susceptible (S), intermediate (I) and resistant (R), n/a, not available.

### Characterization of enterococci

The enterococci isolated from faecal samples were confirmed to be *Enterococcus* spp. by PCR using primers Ent-ES-211-233-F (5′-GHACAGAAGTRAAATAYGAAGG-3′) and Ent-EL-74-95-R (5′-GGNCCTAABGTHACTTTNACTG-3′) and 130 select isolates representing both erythromycin susceptible and resistant categories were further analyzed for species identification by pyrosequencing as described by Zaheer et al. (2012). Thirty six select isolates were subject to PFGE profiling using *Sma*I restriction enzyme using an adaptation of the procedure of Turabelidze et al. (2000). Briefly, bacteria from overnight brain-heart infusion-agar (BHI-agar) cultures were harvested using sterile swabs and suspended in cell suspension buffer [100 mM Tris-HCl (pH 8.0) and 100 mM EDTA], to an optical density (OD) of 1.2–1.3 at 610 nm (1-cm light path). Aliquots (1 ml each) of the suspensions were centrifuged (10,000 × g) for 2 min. in a microcentrifuge and 2/3rd of the supernatant was removed from the tube. The bacterial pellet was resuspended in the remaining supernatant, concentrating the cell suspension to an OD_610_ of 3.6–4.0 (ca. 2.5 × 10^9^ CFU/ml). An aliquot (100 μL) of cell suspension was added to an equal volume of lysis buffer (50 mM Tris-HCl (pH 8), 50 mM EDTA, 625 U/ml mutanolysin, 2.5 mg/ml lysozyme, 1.5 mg/ml proteinase K, 20 μg/ml RNase), mixed gently and incubated at 37°C for 10 min. An equal volume of 1.2% molten SeaKem Gold agarose (FMC BioProducts, Rockland, Maine) containing 1% sodium dodecyl sulfate was added, the mixtures were poured in duplicate into 2-cm by 1-cm by 1.5-mm reusable plug molds (Bio-Rad Laboratories, Hercules, CA) and allowed to solidify at room temperature for 10 min. The duplicate plugs were added to a tube containing 1.8 mL of proteolysis solution [0.44 M EDTA (pH 8.0), 1% sarcosyl, 400 μg/ml of proteinase K] and incubated with constant agitation at 300 rpm for 2 h at 55°C. Plugs were washed 3 times for 10 min each in H_2_O (1.8 mL), followed by 3 times for 10 min in TE (1.8 mL) in a thermomixer set at 50°C and 300 rpm. One plug was cut in three equal slices latitudinally and two of the gel slices were pre-incubated in 200 μL of 1X restriction enzyme buffer for 15 min at 30°C. DNA in the plugs was restricted with 50 units of *Sma*I in a 200 μL reaction mixture for 3 h at 25°C. As a reference standard *Xba*I digested *Salmonella* serotype Braenderup (H9812) plugs were prepared as previously described (Klima et al., 2011).

The digested plugs were embedded in 1% SeaKem Gold low-melting temperature agarose (Lonza Canada, Inc., Shawinigan, QC) that was dissolved in 0.5 × TBE (45 mM Tris, 45 mM boric acid, 1 mM EDTA, pH 8.0). Prior to incorporation into the gel, digested plugs were incubated with 200 μl of 0.5 × TBE at room temperature for 20 min. The digested DNA were separated by PFGE using a CHEF DRII device (Bio-Rad Laboratories Ltd., Mississauga, ON, Canada), at 12°C. The voltage was maintained at 6 V/cm for a total of 21 h with switch times of 4–40 s for initial 12.5 h followed by switch times of 1.5–6 for 8.5 h. The gels were run in 0.5 × TBE buffer containing 0.45 mM thiourea. After electrophoresis, gels were stained with ethidium bromide (1 μg/ml) in distilled water for 20 min followed by three 20 min washes with distilled water. Gels were photographed with an AlphaImager gel documentation system (Alpha Innotech Corp., St. Leandro, CA). Fragment analysis was performed with BioNumerics V5.1 software (Applied Maths Inc., Austin, TX).

### Identification of macrolide resistance determinants

Erythromycin-resistant isolates were evaluated for the presence of the commonly found macrolide resistance determinants *erm*(A), *erm*(B), *erm*(C), *erm*(F), *erm*(T), *erm*(X), *mef*(A) (http://faculty.washington.edu/marilynr/) by PCR analyses. For generating PCR template, a single bacterial colony was suspended in 50 μL of TE (10 mM Tris.HCl pH 8.0, 1 mM EDTA pH 8.0) and incubated at 95°C for 5 min followed by centrifugation at 10,000 × g for 5 min. Supernatant (2 μL) was used as template in a 20 μL volume PCR reaction mixture using PCR primers and reaction conditions as described elsewhere (Chen et al., 2007; Szczepanowski et al., 2009). The commercially available HotStarTaq Plus Master Mix Kit (Qiagen Canada, Inc., Mississauga, ON, Canada) was used according to manufacturer's instructions. Plasmids containing corresponding gene fragments previously cloned in our laboratory were used as positive controls. Select PCR fragments amplified from erythromycin resistant isolates originating from the present study were verified by DNA sequencing.

### Statistical analysis

Data were analyzed using commercially available statistical analysis software (SAS System for Windows, release 9.1.3, SAS Institute, Cary, NC). Prevalence of *M. haemolytica* and erythromycin resistance in enterococci were analyzed using logistic methodology within the GLIMMIX procedure of SAS, with treatment in the model and day of sampling treated as a repeated measure. Model adjusted means (LS means back-transformed to original scale) and standard errors were reported and used to estimate the efficacy of the antibiotic treatments for controlling *M. haemolytica*. For all tests, the level of significance was set at *P* < 0.05.

### Ethics statement

Experiments with beef steers were conducted according to the Canadian Council on Animal Care (CCAC) guidelines. The studies were approved by the institutional Animal Care Committee (ACC), Lethbridge Research Centre, Agriculture and Agri-Food Canada, under protocol number 1111. Antibiotics were administered or fed at levels approved by the Canadian Bureau of Veterinary Drugs and recommended by the manufacturer and used in accordance with industry practices.

## Results

### *Mannheimia haemolytica* characterization

A total of 274 suspect *M. haemolytica* isolates were obtained over the duration of the study of which 260 were confirmed by multiplex PCR assay (Alexander et al., 2008). Isolates were obtained from 29 out of the 40 steers used in the experiment. All of the confirmed *M. haemolytica* isolates were serotyped and 160 (1–3 isolates per *Mannheimia* positive animal per sampling event) were tested for antimicrobial susceptibility using the disk diffusion assay, and 65 (one isolate belonging to each of the *M. haemolytica* positive animals for each sampling event) were subjected to PFGE.

**Figure 2:**
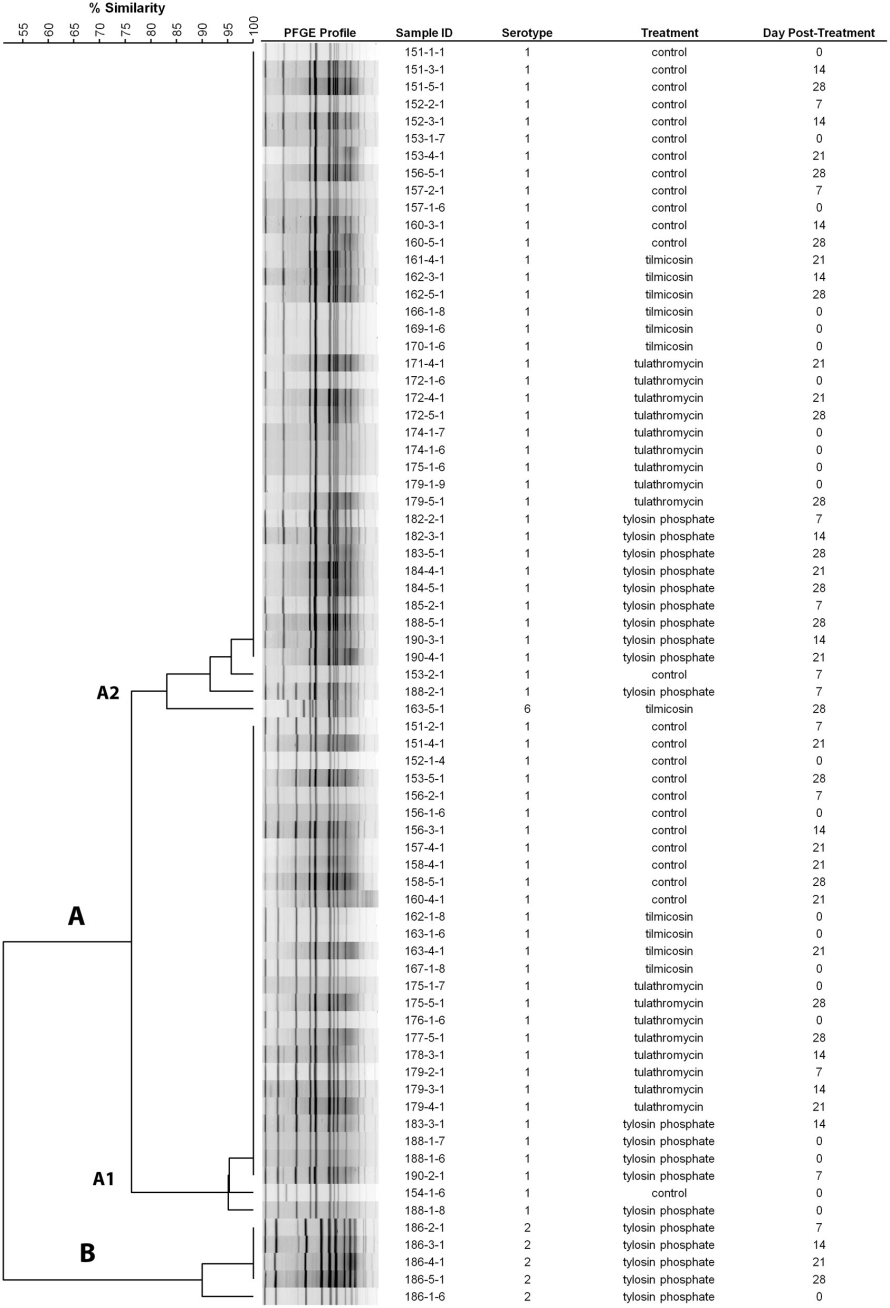
**Dendrogram of PFGE *Sal*I profiles from representative *M. haemolytica* isolates collected weekly.** As an example, sample ID 151-3-1 represents isolate #1 from 3rd sampling event (day 14 post treatment) from animal #151. (Control: animal IDs 151–160; tilmicosin: animal IDs 161–170; tulathromycin: animal IDs 171–180; tylosin: animal IDs 181–190).

Serotyping revealed that 89% (232/260) of the *M. haemolytica* isolates that originated from 93% (27/29) of the positive animals were serotype 1, whereas 8.4% (22/260) of the isolates, all of which originated from a single steer were serotype 2. Two percent (5/260) of the isolates were identified as serotype 6, all of which were obtained from a single steer on the 28th day post treatment. Two main clusters were identified by PFGE analysis, cluster A consisted primarily of serotype 1 isolates with only a single serotype 6 isolate, whereas cluster B was comprised of serotype 2 isolates (Figure 2). Two sub-clusters, A1 and A2 were observed within cluster A; A1 solely comprising serotype 1 isolates and A2 consisting of a mixture of serotype 1 and 6 (Figure 2).

**Figure 3:**
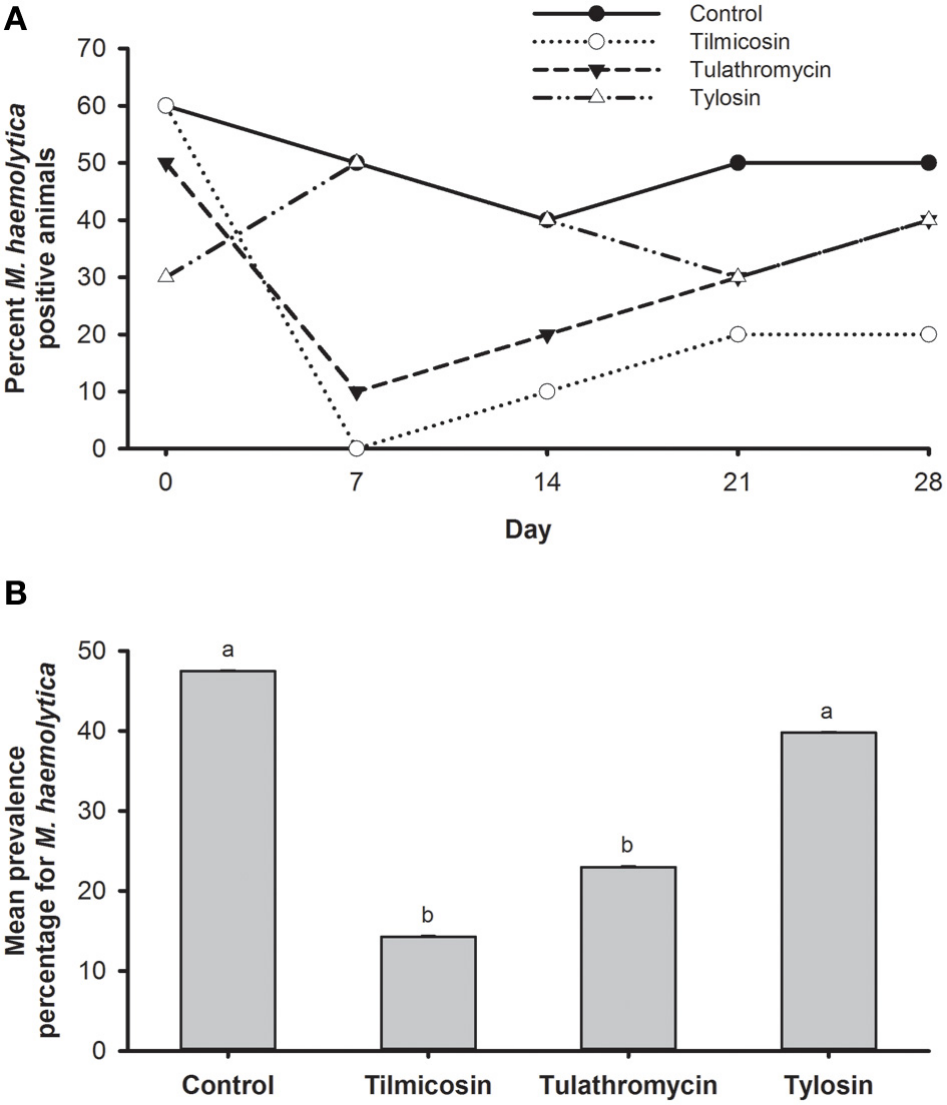
**(A)** Percentage of animals positive for *M. haemolytica* for each of the five sampling events over 28 days study period. **(B)** Mean percentage of *M. haemolytica* prevalence in animals over the entire study period. Means with different superscripts differ (*P* < 0.05).

The prevalence of *M. haemolytica* dropped substantially from steers that were injected with tilmicosin or tulathromycin as compared to levels prior to treatment (Figure 3A). Compared to the control, the number of steers harboring *M. haemolytica* was reduced (*P* < 0.05) by systemic treatment with either tilmicosin or tulathromycin over the post treatment sampling period (days 7–28). Compared to the in-feed tylosin, tilmicosin and tulathromycin also resulted in a reduction (*P* < 0.05) in number of cattle positive for *M. haemolytica* (64 and 42%, respectively) (Figure 3B). In disk diffusion assays, *M. haemolytica* isolates cultured without erythromycin on primary isolation were all sensitive to amoxicillin/clavulanic acid, ampicillin, ceftiofur, danofloxacin, erythromycin, florfenicol, gentamicin, oxytetracycline, spectinomycin, trimethoprim/sulfamethoxazole, tilmicosin, and tulathromycin.

### Characterization of bacteria co-isolated with *M. haemolytica*

**Figure 4:**
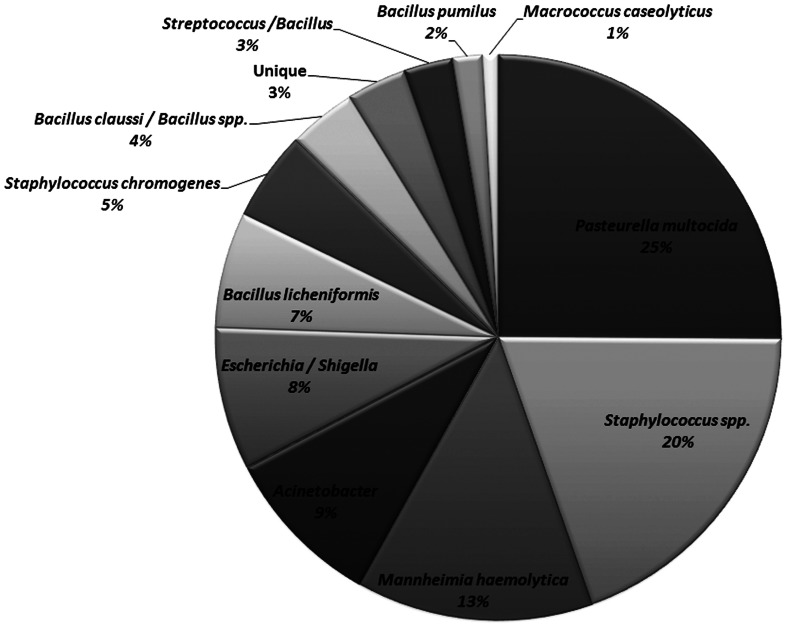
**Relative abundance of bacitracin resistant bacterial species isolated from nasopharynx over 28-days sampling period.** One representative of each morphology per animal per sampling event were used to determine bacterial species prevalence (*n* = 466).

The non-*Mannheimia* bacterial colonies originating from nasopharyngeal samples cultured on BAC or BAC+Ery agar plates were divided into 11 morphological groups (Table 2). The 16S rRNA gene sequences from 165 select isolates with 5–15 isolates representing each morphology group were subsequently analyzed for genus/species identification through alignments using “seqmatch” (http://rdp.cme.msu.edu/index.jsp) or BLAST (http://blast.ncbi.nlm.nih.gov/). The morphotypes were found to be very consistent with 16S rDNA sequence based bacterial identification and therefore were used to define the identity of collected isolates. One representative of each morphotype per sampling event was used to determine bacterial prevalence. Along with *M. haemolytica* (13%), other bacteria found in abundance included *Pasteurella multocida* (25%), *Staphylococcus* spp. (25%), *Acinetobacter* spp. (9%), *E. coli*/*Shigella* group (8%), and *Bacillus licheniformis* (7%) (Figure 4). Among the *Staphylococcus* spp., *S. epidermidis*, *S. pasteuri*, and *S. cohnii* were abundant and collectively constituted 20% of isolated nasopharyngeal bacterial species, with *S. sciuri* only occasionally isolated. These *Staphylococcus* species had indistinguishable colony morphologies and thus collectively constituted one morphological group (Table 2). *Staphylococcus chromogenes* was placed in a separate morphological group due to its distinct yellow color and it constituted 5% of isolated nasopharyngeal bacterial species. Other species such as streptococci, *Macrococcus casseolyticus* and *Bacillus* spp. including *B. clausii* and *B. pumilus* were less abundant (Figure 4). However, collectively and irrespective of morphotypes, *Bacillus* spp. constituted ~14% of isolated bacteria.

**Table 2:** **Bacteria co-isolated with *M. haemolytica***.

**Morphotypes**	**Morphology on BAC-agar plates**	**Species identification based on 16S rDNA sequencing**	**Ery resistance**	**Resistance determinant(s)**
Mh	Small, glossy, grey, beta-haemolytic	*Mannheimia haemolytica*^a^	–	–
1	Small/small-medium, round, glossy, white	*S. epidermidis, S. cohnii, S. pasteuri, S. saprophyticus, S. sciuri*	+	*erm*(C), *erm*(C), *erm*(C), ND, ND
2	Large, mucoid, semi-transparent, grey-white	*Pasteurella multocida*	–	–
3	Very small, dense, brown/pale, alpha-haemolytic	*Streptococcus/Bacillus*	+	ND
4	Medium/large, wrinkly, crusty, fluid-filled, beta-haemolytic	*Bacillus licheniformis*	+	ND
5	Medium/large, glossy, grey-white, mostly beta-haemolytic	*Escherichia coli/Shigella*	+	–
6	Medium/large, rough edges, flat, granular	*Bacillus clausii/Bacillus spp.*	+	ND
7	Small, glossy, grey/cream	*Acinetobacter lwoffii, Acinetobacter spp.*	+	ND
8	Small/medium, glossy, pale-yellow	*Staphylococcus chromogenes*	NA	ND
9	Small/medium, pale-yellow, concentric with concave center	*Macrococcus caseolyticus*	NA	ND
10	Medium, yellowish, concentric circles, very haemolytic, greenish	*Bacillus pumilus*	NA	ND
U	Unique morphologies found occasionally	*Klebsiella*, *Neisseria* spp., *Paenibacillus* spp., uncultured bacteria	NA	ND

a Also confirmed by multiplex PCR assay (Alexander et al., 2008); NA, not available, as those morphology groups were less commonly found on BAC plates and not found on BAC+Ery plates; ND, not determined (no positives detected in PCRs with any of the tested macrolide primer sets, see Materials and Methods).

Erythromycin resistance was found in all isolated bacterial species except *M. haemolytica*, *P. multocida* and other less frequently isolated bacteria (Table 2). Of the seven macrolide resistance genes tested by PCR, the *erm*(C) was predominantly found in *Staphylococcus* spp. (Table 2). With regard to other erythromycin-resistant isolates, no resistance determinants matching any of the seven PCR primer pairs were amplified and therefore were considered as “not detected”.

### Evaluation of enterococci pre- and post-macrolide-treatment

**Figure 5:**
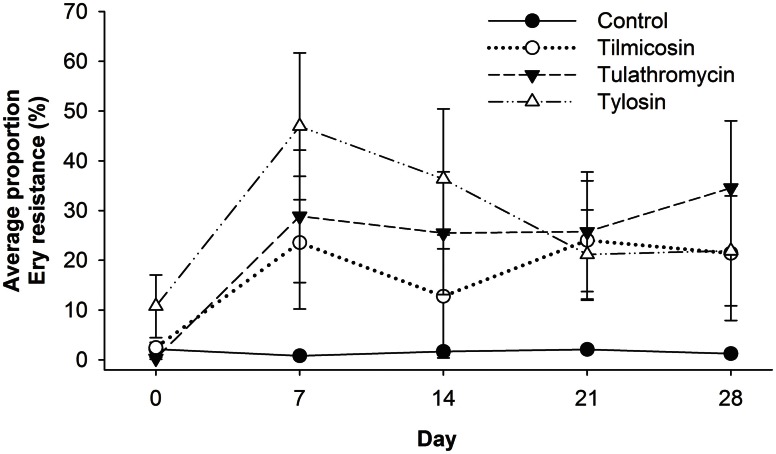
**Proportion of erythromycin-resistant faecal enterococci isolates for each of the five sampling events over 28 days study period, with day 0 samples collected prior to antibiotic treatment.** From day 7 onward, Control isolates had less resistance detected (*P* < 0.05) than antibiotic treated groups, while resistance noted with injectable macrolides (tilimicosin and tulathormycin) did not differ from that cattle fed tylosin.

Enterococci were isolated from all 40 animals (control and treatment groups) on arrival (day 0), and weekly thereafter for 4 weeks (day 7, 14, 21, and 28). Enterococci colonies obtained on BEA and BEA+Ery plates were enumerated and the proportion of erythromycin resistant colonies was calculated for each sample (Figure 5). Compared to the control group, antibiotic treatment groups were 76 times more likely (*P* < 0.02) to have erythromycin resistant enterococci over the study period (days 7–28). Similarly, for all treatment groups, post-treatment samples from the entire study period were 66 times more likely to have erythromycin resistant enterococci (*P* < 0.001) when compared to pre-treatment (day 0) samples. No significant differences in the incidence of erythromycin resistance were observed between injectable (tilmicosin and tulathromycin) and in-feed (tylosin) macrolides.

Speciation of 130 of the enterococci isolates collected from day 0, 7, or 28 revealed that all were *Enterococcus hirae* with the exception of two which were *Enterococcus casseliflavus*. Fifty select isolates of erythromycin resistant enterococci from day 0 and day 7 sampling events were used for the identification of erythromycin resistance determinants. Of the seven macrolide resistance genes investigated via PCR only the *erm*(B) gene was identified in enterococci isolates.

**Figure 6:**
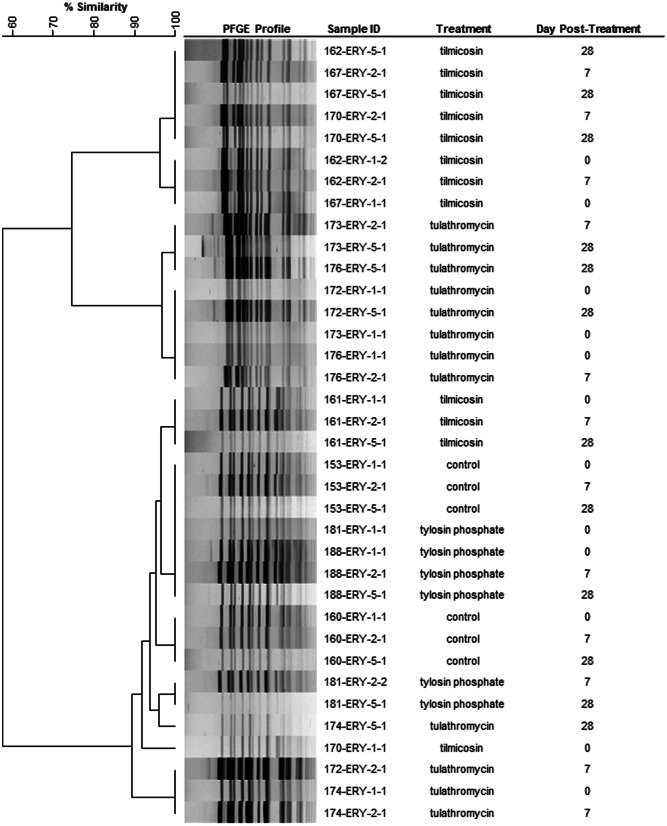
**Dendrogram of PFGE *Sma*I profiles from representative erythromycin resistant enterococci from day 0, 7, and 28.** As an example, sample ID 153-ERY-5-1 represents isolate #1 collected from BEA+ERY plates on 5th sampling event (day 28) from animal #153. (Control: animal IDs 151–160; tilmicosin: animal IDs 161–170; tulathromycin: animal IDs 171–180; tylosin: animal IDs 181–190.

A representative 36 erythromycin resistant enterococci from 12 select animals from three sampling events (day 0, 7, and 28) were subject to PFGE and produced three closely related clusters (>85% similarity) (Figure 6). In eight of the twelve animals the PFGE profiles from the three sampling events had >90% similarity indicating that erythromycin resistant enterococci likely consisted of a persistent clonal population.

## Discussion

The objective of this study was to evaluate and compare the effect of in-feed subtherapeutic and injectable therapeutic administration of macrolides on antimicrobial susceptibility of indicator bacteria from the digestive and respiratory tract of feedlot cattle. For this purpose we selected faecal enterococci and *M. haemolytica* as our indicator bacteria for the digestive and respiratory tract, respectively. Injectable macrolide antimicrobials such as tilmicosin and tulathromycin are commonly used at therapeutic levels in beef cattle production to prevent and treat BRD. The macrolide, tylosin is frequently administered in-feed at subtherapeutic doses for improving feed efficiency and for the reduction of liver abscesses caused by *Fusobacterium necrophorum* and *Actinomyces pyogenes* (www.merckvetmanual.com). The subtherapeutic administration of antibiotics has been hypothesized to promote resistance development as bacteria are exposed to sub-lethal concentrations of antibiotic for prolonged periods of time. Consequently, the subtherapeutic administration of antibiotics in animal feeds for growth promotion has been proposed as a serious public health concern (Aarestrup and Wegener, 1999; Wegener et al., 1999; McEwen and Fedorka-Cray, 2002). Over the last 6–10 years the minimum inhibitory concentrations (MICs) of tilmicosin and tulathromycin towards *M. haemolytica* have markedly increased (Portis et al., 2012), questioning the continued effectiveness of these antibiotics against the etiological agents of BRD. Furthermore, *M. haemolytica* could serve as a reservoir of macrolide resistance genes, potentially disseminating them to other respiratory pathogens.

Deep nasopharyngeal swabs were taken to isolate *M. haemolytica* as this procedure is quick, simple and relatively non-invasive. In our study, a single systemic administration of therapeutic levels of either tilmicosin or tulathromycin was effective in lowering *M. haemolytica* in the nasopharynx of steers (Figures 3A,B). According to the manufacturer, (ELANCO Animal Health, Guelph, On, Canada, www.elanco.ca) injecting cattle with 10 mg of tilmicosin /kg of body weight results in lung concentrations exceeding the MIC(3.12 μg/mL) for *M. haemolytica* for at least 3 days, eliminating it from the respiratory tract for up to 6 days (Frank et al., 2000). Tulathromycin is believed to accumulate in neutrophils and alveolar macrophages (Siegel et al., 2004; Cox et al., 2010), with peak lung levels of 4.1 μg/mL occurring in cattle 24 h after a single injection and concentrations remaining above the MIC for *M. haemolytica* (2.0 μg/mL) for 10 days (www.pfizer.ca). Our data demonstrated that 7 days post-injection, *M. haemolytica* was detected in only one steer treated with tulathromycin and none of the steers treated with tilimicosin, whereas 60% of steers were positive for this bacterium upon arrival (day 0). This suggests that *M. haemolytica* in newly arrived cattle were not macrolide resistant, an observation supported by our inability to isolate erythromycin resistant *M. haemolytica* and likely a reflection of the fact that these cattle had no previous exposure to macrolides.

In contrast to injectable macrolides, tylosin had no effect on the number of *M. haemolytica* in steers administered this antibiotic in feed as compared to cattle that received no antibiotics (Figures 3A,B). This observation likely reflects differences in the method and concentration of the antibiotic administered and the sensitivity of *M. haemolytica* to tylosin. Tylosin is not effective at penetrating the outer membrane of Gram-negative bacteria and as a result its MIC (64 μg/mL) against *M. haemolytica* is much higher than either tilimicosin or tulathromycin (Andersen et al., 2012). Tylosin is known to be widely distributed in body fluids and tissues, but comparative pharmacokinetics of its distribution in the digestive tract relative to the respiratory tract when it is administered in feed are poorly characterized (Lewicki, 2006).

*Mannheimia haemolytica* isolated from animals belonging to control and all three macrolide treatment groups throughout the course of study were found to be susceptible to all tested macrolides indicating that both therapeutic and subtherapeutic administration did not contribute to macrolide resistance in *M. haemolytica* during the study. *Mannheimia haemolytica* isolated in the present study were also susceptible to all other antibiotics tested (Table 2). While tilmicosin and tulathromycin are generally effective against *M. haemolytica*, a few isolates originating from Germany, Japan and United States have shown resistance to these antibiotics (Katsuda et al., 2009; Watts and Sweeney, 2010; Michael et al., 2012). An integrative conjugative element (ICE) has been identified in *P. multocida* that exhibits high similarity to ICEs in *P. multocida* 36950, *Histophilus somni* 23364 and an ICE fragment within the incomplete *M. haemolytica* PHL23 genome (Michael et al., 2012). This is a matter of concern as *P. multocida*, *M. haemolytica*, and *H. somni* often share the same ecological niche in the bovine respiratory tract and exchange of this ICE element could lead to macrolide resistance in these BRD pathogens as transfer of this element among *P. multocida*, *M. haemolytica*, and *E. coli* has been demonstrated in the laboratory (Dabo et al., 2007; Watts and Sweeney, 2010).

The majority of *M. haemolytica* isolates collected from asymptomatic animals in this study were serotype 1 (Figure 2). The PFGE profiles of serotype 1 and 6 isolates clustered together with a ~83% relatedness, a relationship observed previously for these serotypes (Klima et al., 2011). The predominance of a single serotype in the majority of steers is likely a reflection of sourcing them from the same isolated ranch in southern Alberta and transporting them directly to the Lethbridge Research Centre. This arrangement was necessary to ensure that steers had no exposure to antibiotics prior to arrival. Serotype 1 has frequently been linked to clinical disease and both serotypes 1 and 6 are often isolated from cattle with BRD (Zecchinon et al., 2005). However, none of the steers in the present study exhibited clinical BRD. The BRD complex consists of a bacteria (*M. haemolytica, Pasteurella multocida, Histophulus somni, Mycoplasma bovis*) and viruses (Bovine Viral Diarrhea, Infectious Bovine Rhinotracheitis, Bovine Respiratory Synctial Virus, Parainfluenza Type-3 Virus) which together supress innate immune responses and cause lung damage (Fulton, 2009; Pardon et al., 2011). It seems likely that in this study the steers lacked the infectious complex necessary for the development of BRD.

*Pasturella multocida* was the most ubiquitous bacterium isolated from the nasopharynx of steers and in contrast to *M. haemolytica*, prevalence of this bacterium was similar in pre- and post-treatment groups. However, none of the isolated *P. multocida* exhibited resistance to erythromycin. As *P. multocida* were not enumerated, it is possible that macrolides reduced the population of this bacterium. After *P. multocida*, *Staphylococcus* spp. was most abundant with a relative abundances of *S. epidermidis* > *S. pasteuri* > *S. cohnii* > *S. sciuri* > *S. saprophyticus*. Staphylococci are ubiquitous Gram-positive bacteria and are common in the microflora of skin and mucosal surfaces. Currently, there are 31 species recognized in the genus *Staphylococcus* and about half of these are indigenous to humans (Kloos and Bannerman, 1994). The role of some species, such as *S. epidermidis*, *S. saprophyticus*, *S. Pasteuri* and *S. cohni* in human disease has been well documented (Piette and Verschraegen, 2008; Savini et al., 2009). Consistent with previous studies (Aarestrup et al., 2000a; Simeoni et al., 2008), the majority of the erythromycin resistant *Staphylococcus* spp. harbored *erm*(C).

Thirteen percent of the isolated nasopharyngeal bacteria were *M. haemolytica* followed by *Acinetobacter*, Enterobacteriaceae (*Esherichia spp., Shigella spp.)* and *Bacillus* spp. including *B. licheniformis* and *B. clausii*. Most of these bacteria are recognized as part of normal flora of the skin, oropharynx and perineum of healthy individuals. While erythromycin resistance was found in the majority of isolated nasopharyngeal bacteria, macrolide determinants could only be detected in the *Staphylococcus* group. The primers we used to detect seven macrolide resistance genes would not be expected to capture all of the genes potentially conferring resistance. For example, primers for erythromycin resistance genes *erm*(D) and *erm*(34) previously characterized from *B. licheniformis* and *B. clausii*, respectively (Israeli-Reches et al., 1984; Bozdogan et al., 2004) were not included in our panel. Although we observed no increase in the diversity of erythromycin resistant nasopharyngeal bacteria in our study, determinants in those bacteria that possessed them could be disseminated into the broader environment.

*Escherichia coli* are commonly used as faecal indicator bacteria to assess AMR, but we chose enterococci as *E. coli* are intrinsically resistant to macrolides (Mao and Putterman, 1968). Enterococci are common inhabitants of the normal gut flora of both livestock and humans (Yost et al., 2011). Outside of their normal habitat, enterococci are viewed as pathogens and may present a public health concern as they can be transmitted to humans from other hosts or by ingestion of contaminated food or water (Heuer et al., 2006; Marshall and Levy, 2011). Enterococci, in particular *E. faecalis* and *E. faecium* are recognized as prevalent nosocomial pathogens (Fisher and Phillips, 2009; van Schaik and Willems, 2010) with many isolates being resistant to multiple antibiotics and capable of exchanging DNA with other bacteria (SchjØrring and Krogfelt, 2011). In the present study we did not isolate either *E. faecalis* or *E. faecium*, with *E. hirae* being the predominant species isolated from cattle, a species infrequently associated with hospital infections.

The present study revealed a significant increase in the proportion of erythromycin resistant enterococci following macrolide treatment regardless of the method of administration (Figure 5). Oral administration of tylosin was expected to have a direct impact on the enterococci population of the gut, but the occurrence of erythromycin resistant enterococci in cattle administered injectable macrolides was equally marked. Studies submitted to the Food and Drug Administration's Center for Veterinary Medicine (FDA/CVM) showed that with a single subcutaneous dose of tilmicosin to cattle, 24% was recovered in the urine and 68% in the feces, whereas with tulathromycin, 50% was recovered in the feces with 90% of this being in its original form. It has been proposed that tulathromycin losses activity at pH ≤ 7.0, (Food and Drug Administration's Center for Veterinary, 2013), but considering that the pH in intestinal digesta and in feces is usually neutral or acidic (Allison et al., 1979; Canh et al., 1997), our results would suggest that this antibiotic selected for resistant enterococci within the intestinal tract. It is possible that the forage rich diet used in our study resulted in intestinal contents having, a pH above 7.0, allowing the concentration of tulathromycin in digesta to exceed the MIC of enterococci. High forage diets in cattle are known to increase colonic pH to ranges between 7.4 and 8.0 (Scott et al., 2000; Loy et al., 2001), but results could be quite different on high grain diets where the pH of digesta is considerably lower.

Similar PFGE profiles were observed for erythromycin resistant enterococci from both pre- and post-treatment samples (Figure 6), suggesting that regardless of the method of administration, macrolides selected for erythromycin resistant enterococci that were already in the digestive tract. Selection for resistant enterococci combined with a reduction in susceptible enterococci significantly increased the presence of erythromycin resistant *Enterococcus* spp. within the digestive tract. This observation is of interest considering that the cattle used in this study originated from a very isolated ranch and never had prior direct exposure to macrolides. It would be interesting to examine the persistence of this resistant population for a prolonged period of time to understand population dynamics and to investigate if the metabolic burden/cost of antibiotic resistance genes in the absence of macrolides leads to a decline in resistance within the enterococci population over time. As is typical in industry, cattle in this study were treated with injectable macrolides early in the feeding period. Considering that they would have been fed for an additional 200 days prior to slaughter, loss of resistant enterococci from the intestinal tract at later points in the feeding period is a distinct possibility. However, resistant enterococci may persist in cattle fed tylosin as this antibiotic is often administered for a longer duration of the feeding period.

All of the isolated erythromycin resistant enterococci contained *erm*(B), a gene coding for rRNA adenine N-6-methyltransferase, which methylates the A2058 position of 23S rRNA. Macrolide resistance in enterococci isolates from humans and animal sources in Europe has been well documented (Jensen et al., 1999; Aarestrup et al., 2001). Occurrence of macrolide resistance in enterococci originating from swine is thought to stem from the subtherapeutic use of tylosin (Jackson et al., 2004). The co-existence of macrolide resistance genes with other antibiotic resistance genes has also been observed, most notably a link between resistance to macrolides and vancomycin (Aarestrup et al., 2000b), attributable to *erm*(B) and the *vanA* gene occurring in close proximity on the same plasmid. The increased occurrence of both *erm* and *tet* (tetracycline resistance) genes in faecal microbial communities from beef cattle fed subtherapeutic levels of tylosin has also been identified (Chen et al., 2008). Linkage of determinants for MLS_B_ and chloramphenicol resistance has also been found on a single conjugative plasmid in *E. faecium* and dissemination of this cluster among streptogramin-resistant enterococci occurs (Werner et al., 2000). Resistance to MLS_B_ antibiotics in Gram-positive cocci colonizing humans is now recognized to be a serious problem, negatively affecting clinical outcomes (Lim et al., 2002; DiPersio and DiPersio, 2006).

Regardless of the hypothesized prospects of subtherapeutic administration of antimicrobials contributing towards AMR development, there is limited and conflicting data as to the extent that subtherapeutic vs. therapeutic drug administration contributes to livestock mediated antimicrobial resistance. Studies have shown that subtherapeutic administration of tylosin had no impact on the prevalence of erythromycin resistant *Campylobacter* in feedlot cattle (Inglis et al., 2005), whereas with broiler chickens the frequency of macrolide resistant *Campylobacter* in cecal contents was increased with subtherapeutic vs. therapeutic doses of tylosin (Ladely et al., 2007). These discrepancies may reflect species-specific (cattle vs. chicken) differences in gastrointestinal physiology and diet. Others have found that short-term therapeutic use of chlortetracycline in the diet was no less likely to select for resistant *Salmonella* populations than long-term subtherapeutic use (Kobland et al., 1987). In-feed and subcutaneous administration of oxytetracycline were also equally responsible for increasing the proportion of feedlot cattle excreting tetracycline resistant *E. coli* in faeces (Checkley et al., 2010).

The present study offers a comparison of subtherapeutic and therapeutic drug administration with regards to the prevalence of resistance among bacteria from two independent locations in cattle. In conclusion, the injectable macrolides had impact on both respiratory and enteric microbes whereas orally administered macrolides only influenced enteric bacteria. Therapeutic levels of tilmicosin and tulathromycin were effective in lowering nasopharyngeal *M. haemolytica*, whereas the in-feed levels of tylosin had no effect on the prevalence of this bacterium. *M. haemolytica* isolates from control and macrolide treated animals were found to be susceptible to macrolides as well as other antibiotics tested. The lack of AMR in *M. haemolytica* may be attributed to the possible absence of AMR determinants in *Mannheimia* as well as other closely related bacteria such as *P. multocida*. Erythromycin resistance was detected in nasopharyngeal bacteria co-isolated with *M. haemolytica*, regardless of the treatment group. All three macrolides increased the occurrence of erythromycin resistance *Enterococcus* spp. within the intestinal tract of cattle, but the species identified were not those most frequently linked to nosocomial infections in humans. To our knowledge this is the first report on increased occurrence of macrolide resistance in enterococci after systemic macrolide usage in cattle. It would be interesting to monitor the post-treatment AMR resistance over a period of weeks to months beyond treatment to determine if these macrolide-resistant enterococci continue to persist within the faecal bacterial populations of cattle.

### Conflict of interest statement

The authors declare that the research was conducted in the absence of any commercial or financial relationships that could be construed as a potential conflict of interest.

## References

[B1] Aarestrup F. M. Agersł Y. Ahrens P. Jłrgensen J. C. Madsen M. Jensen L. B. (2000a). Antimicrobial susceptibility and presence of resistance genes in staphylococci from poultry. Vet. Microbiol. 74, 353–364 10.1016/S0378-1135(00)00197-8 10831857

[B2] Aarestrup F. M. Kruse H. Tast E. Hammerum A. M. Jensen L. B. (2000b). Associations between the use of antimicrobial agents for growth promotion and the occurrence of resistance among *Enterococcus faecium* from broilers and pigs in Denmark, Finland, and Norway. Microb. Drug Resist. 6, 63–70 10868809 10.1089/mdr.2000.6.63

[B3] Aarestrup F. M. Wegener H. C. (1999). The effects of antibiotic usage in food animals on the development of antimicrobial resistance of importance for humans in *Campylobacter* and *Escherichia coli.* Microbes. Infect. 1, 639–644 10.1016/S1286-4579(99)80064-1 10611741

[B4] Aarestrup F. M. A. Seyfarth M. Emborg H. D. Pedersen K. Hendriksen R. S. Bager F. (2001). Effect of abolishment of the use of antimicrobial agents for growth promotion on occurrence of antimicrobial resistance in fecal enterococci from food animals in Denmark. Antimicrob. Agents Chemother. 45, 2054–2059 10.1128/AAC.45.7.2054-2059.2001 11408222 PMC90599

[B5] Addah W. Baah J. Groenewegen P. Okine E. K. McAllister T. A. (2011). Comparison of the fermentation characteristics, aerobic stability and nutritive value of barley and corn silages ensiled with or without a mixed bacterial inoculant. Can. J. Anim. Sci. 91, 133–146

[B6] Alexander T. W. Cook S. R. Yanke L. J. Booker C. W. Morley P. S. Read R. R. (2008). A multiplex polymerase chain reaction assay for the identification of *Mannheimia haemolytica, Mannheimia glucosida* and *Mannheimia ruminalis*. Vet. Microbiol. 130, 165–175 10.1016/j.vetmic.2008.01.001 18308486

[B7] Allison M. J. Robinson I. M. Bucklin J. A. Booth G. D. (1979). Comparison of bacterial populations of the pig cecum and colon based upon enumeration with specific energy sources. Appl. Environ. Microbiol. 37, 1142–1151 384906 10.1128/aem.37.6.1142-1151.1979 PMC243369

[B8] Andersen N. M. Poehlsgaard J. Warrass R. Douthwaite S. (2012). Inhibition of protein synthesis on the ribosome comparing tildipirosin with other veterinary macrolides. Antimicrob. Agents Chemother. 56, 6033–6036 10.1128/AAC.01250-12 22926570 PMC3486562

[B9] Bozdogan B. Galopin S. Leclercq R. (2004). Characterization of a new erm-related macrolide resistance gene present in probiotic strains of *Bacillus clausii.* Appl. Environ. Microbiol. 70, 280–284 10.1128/AEM.70.1.280-284.2004 14711653 PMC321311

[B10] Canh T. T. Verstegen M. W. A. Aarnink A. J. A. Schrama J. W. (1997). Influence of dietary factors on nitrogen partitioning and composition of urine and feces of fattening pigs. J. Anim. Sci. 75, 700–706 9078486 10.2527/1997.753700x

[B11] Checkley S. L. Campbell J. R. Chirino-Trejo M. Janzen E. D. Waldner C. L. (2010). Associations between antimicrobial use and the prevalence of antimicrobial resistance in fecal *Escherichia coli* from feedlot cattle in western Canada. Can. Vet. J. 51, 853–861 21037885 PMC2905004

[B12] Chen J. Fluharty F. L. St-Pierre N. Morrison M. Yu Z. (2008). Technical note: occurrence in fecal microbiota of genes conferring resistance to both macrolide-lincosamide-streptogramin B and tetracyclines concomitant with feeding of beef cattle with tylosin. J. Anim. Sci. 86, 2385–2391 10.2527/jas.2007-0705 18469042

[B13] Chen J. Yu Z. Michel F. C. Jr. Wittum T. Morrison M. (2007). Development and application of real-time PCR assays for quantification of *erm* genes conferring resistance to macrolides-lincosamides-streptogramin B in livestock manure and manure management systems. Appl. Environ. Microbiol. 73, 4407–4416 10.1128/AEM.02799-06 17496134 PMC1932836

[B14] CIPARS. (2013). Canadian Integrated Program for Antimicrobial Resistance Surveillance Report. Available online at: http://www.phac-aspc.gc.ca/cipars-picra/pubs-eng.php#ar (last accessed date: 8 March 2013).

[B15] Clinical Laboratory Standards Institute (2008a). Performance Standards for Antimicrobial Disk and Dilution Susceptibility Tests for Bacteria Isolated from Animals; Approved Standard - 3rd Edn. Document M31-A3. Wayne, PA: Clinical and Laboratory Standards Institute

[B15a] Clinical Laboratory Standards Institute (2008b). Methods for Antimicrobial Dilution and Disk Susceptibility Testing of Infrequently Isolated or Fastidious Bacteria; Approved Guideline. Document M45-A. Wayne, PA: Clinical and Laboratory Standards Institute

[B16] Confer A. W. (2009). Update on bacterial pathogenesis in BRD. Anim. Health Res. Rev. 10, 145–148 10.1017/S1466252309990193 20003651

[B17] Cox S. R. McLaughlin C. Fielder A. E. Yancey M. F. Bowersock T. L. Garcia-Tapia D. (2010). Rapid and prolonged distribution of tulathromycin into lung homogenate and pulmonary epithelial lining fluid of Holstein calves following a single subcutaneous administration of 2.5 mg/kg body weight. Int. J. Appl. Res. Vet. Med. 8, 129–137

[B18] Dabo S. M. Taylor J. D. Confer A. W. (2007). *Pasteurella multocida* and bovine respiratory disease. Anim. Health Res. Rev. 8, 129–150 10.1017/S1466252307001399 18218157

[B19] Desmolaize B. Rose S. Warrass R. Douthwaite S. (2011). A novel *Erm* monomethyltransferase in antibiotic-resistant isolates of *Mannheimia haemolytica* and *Pasteurella multocida.* Mol. Microbiol. 80, 184–194 10.1111/j.1365-2958.2011.07567.x 21371136

[B20] DiPersio L. P. DiPersio J. R. (2006). High rates of erythromycin and clindamycin resistance among OBGYN isolates of group B *Streptococcus.* Diagn. Microbiol. Infect. Dis. 54, 79–82 10.1016/j.diagmicrobio.2005.07.003 16368478

[B21] Fisher K. Phillips C. (2009). The ecology, epidemiology and virulence of *Enterococcus.* Microbiol. 55, 1749–1757 10.1099/mic.0.026385-0 19383684

[B22] Food Drug Administration's Center for Veterinary Medicine (FDA/CVM). (2013). Tulathromycin solution for parenteral injection for treatment of bovine and swine respiratory diseases: microbiological effects on bacteria of human health concern, a qualitative risk estimation. Available online at: www.fda.gov/downloads/AdvisoryCommittees/CommitteesMeetingMaterials/VeterinaryMedicineAdvisoryCommittee/UCM127196.pdf (last accessed date: 8 March 2013).

[B23] Frank G. H. Briggs R. E. Loan R. W. Purdy C. W. Zehr E. S. (2000). Effect of tilmicosin treatment on *Pasteurella haemolytica* organisms in nasal secretion specimens of calves with respiratory tract disease. Am. J.Vet. Res. 61, 525–529 10803647 10.2460/ajvr.2000.61.525

[B24] Fulton R. W. (2009). Bovine respiratory disease research (1983–2009). Anim. Health Res. Rev. 10, 131–139 10.1017/S146625230999017X 20003649

[B25] Giraffa G. (2002). Enterococci from foods. FEMS Microbiol. Rev. 26, 163–171 12069881 10.1111/j.1574-6976.2002.tb00608.x

[B26] Gow S. (2005). Antimicrobial resistance, prudent use, and the Canadian integrated program for antimicrobial resistance surveillance (CIPARS). Large Anim. Vet Rounds 5, 1–6 10.1111/j.1863-2378.2010.01356.x 21083820

[B27] Heuer O. E. Hammerum A. M. Collignon P. Collignon P. Wegener H. C. (2006). Human health hazard from antimicrobial-resistant enterococci in animals and food. Clin. Infect. Dis. 43, 911–916 10.1086/507534 16941376

[B28] Inglis G. D. McAllister T. A. Busz H. W. Yanke L. J. Morck D. W. Olson M. E. (2005). Effects of subtherapeutic administration of antimicrobial agents to beef cattle on the prevalence of antimicrobial resistance in *Campylobacter jejuni* and *Campylobacter hyointestinalis.* Appl. Environ. Microbiol. 71, 3872–3881 10.1128/AEM.71.7.3872-3881.2005 16000800 PMC1169002

[B29] Israeli-Reches M. Weinrauch Y. Dubnau D. (1984). Evolutionary relationships of the *Bacillus licheniformis* macrolide-lincosamide-streptogramin B resistance elements. Mol. Gen. Genet. 194, 362–367 6429478 10.1007/BF00425545

[B30] Jackson C. R. Fedorka-Cray P. J. Barrett J. B. Ladely S. R. (2004). Effects of tylosin use on erythromycin resistance in enterococci isolated from swine. Appl. Environ. Microbiol. 70, 4205–4210 10.1128/AEM.70.7.4205-4210.2004 15240302 PMC444810

[B31] Jensen L. B. Frimodt-Moller N. Aarestrup F. M. (1999). Presence of *erm* gene classes in gram-positive bacteria of animal and human origin in Denmark. FEMS Microbiol. Lett. 170, 151–158 9919664 10.1111/j.1574-6968.1999.tb13368.x

[B32] Jensen L. B. Hammerum A. M. Bager F. Aarestrup F. M. (2002). Streptogramin resistance among *Enterococcus faecium* isolated from production animals in Denmark in 1997. Microb. Drug Resist. 8, 369–374 10.1089/10766290260469642 12523635

[B33] Katsuda K. Kohmoto M. Mikami O. Uchida I. (2009). Antimicrobial resistance and genetic characterization of fluoroquinolone-resistant *Mannheimia haemolytica* isolates from cattle with bovine pneumonia. Vet. Microbiol. 139, 74–79 10.1016/j.vetmic.2009.04.020 19428195

[B34] Klima C. L. Alexander T. W. Read R. R. Gow S. P. Booker C. W. Hannon S. (2011). Genetic characterization and antimicrobial susceptibility of *Mannheimia haemolytica* isolated from the nasopharynx of feedlot cattle. Vet. Microbiol. 149, 390–398 10.1016/j.vetmic.2010.11.018 21146332

[B35] Kloos W. Bannerman T. L. (1994). Update on clinical significance of coagulase-negative staphylococci. Clin. Microbiol. Rev. 7, 117–140 10.1128/CMR.7.1.117 8118787 PMC358308

[B36] Kobland J. D. Gale G. O. Gustafson R. H. Simkins K. L. (1987). Comparison of therapeutic versus subtherapeutic levels of chlortetracycline in the diet for selection of resistant *salmonella* in experimentally challenged chickens. Poult. Sci. 66, 1129–1137 3313364 10.3382/ps.0661129

[B37] Ladely S. R. Harrison M. A. Fedorka-Cray P. J. Berrang M. E. Englen M. D. Meinersmann R. J. (2007). Development of macrolide-resistant Campylobacter in broilers administered subtherapeutic or therapeutic concentrations of tylosin. J. Food Prot. 70, 1945–1951 17803155 10.4315/0362-028x-70.8.1945

[B38] Lewicki J. (2006). Tylosin. A review of pharmacokinetics, residues in food animals and analytical methods. FAO website: ftp://ftp.fao.org/ag/agn/food/tylosin_2006.pdf (last accessed date: 8 March 2013).

[B39] Lim J. A. Kwon A. R. Kim S. K. Chong Y. Lee K. Choi E. C. (2002). Prevalence of resistance to macrolide, lincosamide and streptogramin antibiotics in Gram-positive cocci isolated in a Korean hospital. J. Antimicrob. Chemother. 49, 489–495 10.1093/jac/49.3.489 11864949

[B40] Loy T. Wilson C. Bailey D. Klopfenstein T. J. Moxley R. A. (2001). Influence of restricted intake and reduced dietary starch on colonic pH and *E. coli* prevalence. Nebraska Beef Report MP76-A, 86–88

[B41] Marshall B. M. Levy S. B. (2011). Food animals and antimicrobials: impacts on human health. Clin. Microbiol. Rev. 24, 718–733 10.1128/CMR.00002-11 21976606 PMC3194830

[B42] Mao J. C. Putterman M. (1968). Accumulation in gram-postive and gram-negative bacteria as a mechanism of resistance to erythromycin. J. Bacteriol. 95, 1111–1117 4966821 10.1128/jb.95.3.1111-1117.1968 PMC252138

[B43] McEwen S. A. Fedorka-Cray P. J. (2002). Antimicrobial use and resistance in animals. Clin. Infect. Dis. 34, S93–S106 11988879 10.1086/340246

[B44] Michael G. B. Kadlec K. Sweeney M. T. Brzuszkiewicz E. Liesegang H. Daniel R. (2012). ICEPmu1, an integrative conjugative element (ICE) of *Pasteurella multocida*: analysis of the regions that comprise 12 antimicrobial resistance genes. J. Antimicrob. Chemother. 67, 84–90 10.1093/jac/dkr406 22001175

[B45] Pardon B. De Bleecker K. Dewulf J. Callens J. Boyen F. Catry B. (2011). Prevalence of respiratory pathogens in diseased, non-vaccinated, routinely medicated veal calves. Vet. Rec. 169, 278 10.1136/vr.d4406 21831999

[B46] Piette A. Verschraegen G. (2008). Role of coagulase-negative staphylococci in human disease. Vet. Microbiol. 134, 45–54 10.1016/j.vetmic.2008.09.009 18986783

[B47] Portis E. Lindeman C. Johansen L. Stoltman G. (2012). A ten-year (2000–2009) study of antimicrobial susceptibility of bacteria that cause bovine respiratory disease complex–*Mannheimia haemolytica, Pasteurella multocida*, and *Histophilus somni*–in the United States and Canada. J. Vet. Diagn. Invest. 24, 932–944 10.1177/1040638712457559 22914822

[B48] Roberts M. C. (2008). Update on macrolide-lincosamide-streptogramin, ketolide, and oxazolidinone resistance genes. FEMS Microbiol. Lett. 282, 147-159 10.1111/j.1574-6968.2008.01145.x 18399991

[B49] Roberts M. C. Sutcliffe J. Courvalin P. Jensen L. B. Rood J. Seppala H. (1999). Nomenclature for macrolide and macrolide-lincosamide-streptogramin B resistance determinants. Antimicrob. Agents Chemother. 43, 2823–2830 10582867 10.1128/aac.43.12.2823 PMC89572

[B50] Savini V. Catavitello C. Carlino D. Bianco A. Pompilio A. Balbinot A. (2009). *Staphylococcus pasteuri* bacteraemia in a patient with leukaemia. J. Clin.Pathol. 62, 957–958 10.1136/jcp.2009.067041 19542075

[B51] SchjØrring S. Krogfelt K. A. (2011). Assessment of bacterial antibiotic resistance transfer in the gut. Int. J. Microbiol. 2011:ID 312956. 10.1155/2011/312956 21318188 PMC3034945

[B52] Schlünzen F. Zarivach R. Harms J. Bashan A. Tocilj A. Albrecht R. (2001). Structural basis for the interaction of antibiotics with the peptidyl transferase centre in eubacteria. Nature 413, 814–821 10.1038/35101544 11677599

[B53] Scott T. Wilson C. Bailey D. Klopfendtein T. J. Milton T. (2000). Influence of diet on total and acid resistant *E. coli* and colonic pH. Nebraska Beef Report MP73-A, 39–43 10.1017/S0007114509992960 20028602

[B54] Siegel T. W. Earley D. L. Smothers C. D. Sun F. Ricketts A. P. (2004). Cellular uptake of the triamilide tulathromycin by bovine and porcine phagocytic cells *in vitro.* J. Anim. Sci. 82, 186

[B55] Simeoni D. Rizzotti L. Cocconcelli P. Gazzola S. Dellaglio F. Torriani S. (2008). Antibiotic resistance genes and identification of staphylococci collected from the production chain of swine meat commodities. Food Microbiol. 25, 196–201 10.1016/j.fm.2007.09.004 17993395

[B56] Skinner R. Cundliffe E. Schmidt F. J. (1983). Site of action of a ribosomal RNA methylase responsible for resistance to erythromycin and other antibiotics. J. Biol. Chem. 258, 12702–12706 6195156

[B57] Szczepanowski R. Linke B. Krahn I. Gartemann K. H. Gützkow T. Eichler W. (2009). Detection of 140 clinically relevant antibiotic-resistance genes in the plasmid metagenome of wastewater treatment plant bacteria showing reduced susceptibility to selected antibiotics. Microbiol. 155, 2306–2319 10.1099/mic.0.028233-0 19389756

[B58] Turabelidze D. Kotetishvili M. Kreger A. Morris J. G. Jr. Sulakvelidze A. (2000). Improved pulsed-field gel electrophoresis for typing vancomycin-resistant enterococci. J. Clin. Microbiol. 38, 4242–4245 11060099 10.1128/jcm.38.11.4242-4245.2000 PMC87572

[B59] USDA. (1999). Report part III: health management and biosecurity in U.S. feedlots 1999. Available online at: http://www.aphis.usda.gov/animal_health/nahms/feedlot/downloads/feedlot99/Feedlot99_dr_PartIII.pdf (last accessed date: 8 March 2013).

[B60] van Schaik W. Willems R. J. (2010). Genome-based insights into the evolution of enterococci. Clin. Microbiol. Infect. 16, 527–532 10.1111/j.1469-0691.2010.03201.x 20569263

[B61] Watts J. L. Sweeney M. T. (2010). Antimicrobial resistance in bovine respiratory disease pathogens: measures, trends, and impact on efficacy. Vet. Clin. North Am. Food Anim. Pract. 26, 79–88 10.1016/j.cvfa.2009.10.009 20117544

[B62] Wegener H. C. Aarestrup F. M. Jensen L. B. Hammerum A. M. Bager F. (1999). Use of antimicrobial growth promoters in food animals and *Enterococcus faecium* resistance to therapeutic antimicrobial drugs in Europe. Emerg. Infect. Dis. 5, 329–335 10.3201/eid0503.990303 10341169 PMC2640785

[B63] Werner G. Hildebrandt B. Klare I. Witte W. (2000). Linkage of determinants for streptogramin A, macrolide-lincosamide-streptogramin B, and chloramphenicol resistance on a conjugative plasmid in *Enterococcus faecium* and dissemination of this cluster among streptogramin-resistant enterococci. Int. J. Med. Microbiol. 290, 543–548 10.1016/S1438-4221(00)80020-X 11100829

[B64] Yost C. K. Diarra M. S. Topp E. (2011). Animals and humans as sources of fecal indicator bacteria, in The Fecal Bacteria, eds Sadowsky M. J. Whitman R. L. (Washington DC: ASM Press), 67–91

[B65] Zaheer R. Yanke L. J. Church D. Topp E. Read R. R. McAllister T. A. (2012). High-throughput species identification of enterococci using pyrosequencing. J. Microbiol. Methods 89, 174–178 10.1016/j.mimet.2012.03.012 22465481

[B66] Zecchinon L. Fett T. Desmecht D. (2005). How *Mannheimia haemolytica* defeats host defence through a kiss of death mechanism. Vet. Res. 36, 133–156 10.1051/vetres:2004065 15720968

